# Down-regulation of PBK inhibits proliferation of human endometrial stromal cells in thin endometrium

**DOI:** 10.1186/s12958-022-00903-8

**Published:** 2022-02-02

**Authors:** Qi Zhu, Simin Yao, Yishan Dong, Dan Liu, Huiyan Wang, Peipei Jiang, Chenyan Dai, Haining Lv, Chenrui Cao, Zhenhua Zhou, Limin Wang, Wenjing Gou, Xiwen Zhang, Guangfeng Zhao, Yali Hu

**Affiliations:** 1 Department of Obstetrics and Gynecology, Nanjing Drum Tower Hospital, Chinese Academy of Medical Science and Peking Union Medical College, Graduate School of Peking Union Medical College, 321 Zhongshan Rd, Nanjing, 210008 China; 2 Department of Obstetrics and Gynecology, The Affiliated Drum Tower Hospital of Nanjing University Medical School, Nanjing, China; 3 Department of Obstetrics and Gynecology, Drum Tower Clinical Medical College, Nanjing Medical University, Nanjing, China

**Keywords:** Thin endometrium, RNA-seq, Transcriptome, PBK, TOPK, HESCs

## Abstract

**Background:**

Thin endometrium (TE) is a challenging clinical issue in the reproductive medicine characterized by inadequate endometrial thickness, poor response to estrogen and no effective treatments currently. At present, the precise pathogenesis of thin endometria remains to be elucidated. We aimed to explore the related molecular mechanism of TE by comparing the transcriptome profiles of late-proliferative phase endometria between TE and matched controls.

**Methods:**

We performed a bulk RNA-Seq (RNA-sequencing) of endometrial tissues in the late-proliferative phase in 7 TE and 7 matched controls for the first time. Differential gene expression analysis, gene ontology enrichment analysis and protein-protein interactions (PPIs) network analysis were performed. Immunohistochemistry was used for molecular expression and localization in endometria. Human endometrial stromal cells (HESCs) were isolated and cultured for verifying the functions of hub gene.

**Results:**

Integrative data mining of our RNA-seq data in endometria revealed that most genes related to cell division and cell cycle were significantly inhibited, while inflammation activation, immune response and reactive oxygen species associated genes were upregulated in TE. PBK was identified as a hub of PPIs network, and its expression level was decreased by 2.43-fold in endometria of TE patients, particularly reduced in the stromal cells, which was paralleled by the decreased expression of Ki67. In vitro experiments showed that the depletion of PBK reduced the proliferation of HESCs by 50% and increased the apoptosis of HESCs by 1 time, meanwhile PBK expression was inhibited by oxidative stress (reduced by 76.2%), hypoxia (reduced by 51.9%) and inflammatory factors (reduced by approximately 50%). These results suggested that the insufficient expression of PBK was involved in the poor endometrial thickness in TE.

**Conclusions:**

The endometrial transcriptome in late-proliferative phase showed suppressed cell proliferation in women with thin endometria and decreased expression of PBK in human endometrial stromal cells (HESCs), to which inflammation and reactive oxygen species contributed.

**Supplementary Information:**

The online version contains supplementary material available at 10.1186/s12958-022-00903-8.

## Introduction

A successful pregnancy requires high-quality embryos and well receptive endometria [1]. Endometrial thickness and pattern have been widely regarded as a basic index reflecting the endometrial receptivity [2]. With the decrease of endometrial thickness, the embryo implantation and pregnancy rate reduce [3]. Thin endometrium (TE) is often considered as endometrial thickness < 7 mm in mid-luteum and poor response to estrogen stimulation [4].

The main clinical characteristics of TE patients are normal menstrual cycle but too little menstrual volume, decreased pregnancy rate and recurrent pregnancy loss [5, 6]. TE is often secondary to curettage and surgical separation of intrauterine adhesions. Several adjuvant regimens have been proposed for treatment, including the administrations of low-dose aspirin [7, 8], estradiol [9, 10], sildenafil citrate [11, 12], granulocyte colony-stimulating factor (G-CSF) [13, 14] and tocopherol (vitamin E) [15]. However, there is no sufficient evidence that any of the above adjuvants is effective for patients with TE [16]. Since few reports are related to the molecular mechanisms of TE, which hinders the development of therapeutic methods, it is urgently needed to reveal the pathogenesis of TE.

In this study, we performed an RNA-seq analysis of endometrial tissue in the late-proliferative phase in TE and matched controls and found that the endometria of TE patients mainly presented downregulation in cell cycle and proliferation relative genes and upregulation in inflammation and reactive oxygen species relative genes. We demonstrated that the downregulation of PDZ-binding kinase (PBK) was involved in the poor endometrial development in TE patients.

## Materials and methods

### Human endometrial samples

This study was approved by the Ethics Committee of Nanjing Drum Tower Hospital, The Affiliated Hospital of Nanjing University Medical School (No.2016-129-01). All participants signed informed consent forms before the endometrial biopsy was performed. The endometria were collected from 7 patients with thin endometrium and 7 controls with normal thickness endometrium for the whole transcriptome expression profiles. In parallel, endometria from 19 controls and 29 TE patients were additionally collected for further validation of hub gene expression by qRT-PCR and immunohistochemistry. Human endometrial samples were collected during the late-proliferative phase of the menstrual cycle from women of childbearing age during hysteroscopic screening for infertility. Late proliferative phase was based on the low serum progesterone level and follicular diameter of 15 ~ 17 mm. Thickness of endometrium was measured by vaginal ultrasonography. The infertile patients with normal endometrium (endometrial thickness > 8 mm in mid-luteum) and normal ovary function were recruited as the control group. Diagnosis of TE was based on endometrium thickness < 7 mm in mid-luteum in previous cycles. Clinical information of all donors is summarized in Supplementary Table S1.

A total of 62 women of child-bearing age participated in the study, including 36 patients with thin endometrium and 26 controls with normal thickness endometrium.

### HESCs isolation, culture, and in vitro drug treatment

HESCs were isolated and cultured by procedures described previously [17].

For in vitro stimulation experiments, HESCs were cultured in 2% FBS-DMEM/F12 medium under the conditions of 21% O_2_, 5% CO_2_ and 37 °C, containing TGFβ1 (10 ng/ml, PeproTech, cat# 100–21, Rocky Hill, NJ, USA) or IL-1β (10 ng/ml) or TNFα (10 ng/ml) for 24 h and 48 h, respectively. The hypoxia experiment was carried out by culturing the cells in 1% O_2_, 5% CO_2_ and 37 °C hypoxia incubator for 24 h and 48 h. For the gene silencing of PBK, HESCs were cultured in opti-MEM (Gibco, USA) and transfected with Lipofectamine 2000 at approximately 50% confluence according to the manufacturer’s guidelines (Invitrogen, USA). Three siRNAs against PBK and negative control (si-NC) were designed and constructed by RiboBio (Guangzhou, China). The sequence information is as follows:

- siPBK 1 (si-1): 5′-GAACTAGGCCACCTATTAA-3′,
- siPBK 2 (si-2): 5′-GAATCATACCAGAAAGTAA-3′,
- siPBK 3 (si-3): 5′-GAGACATAAAGTCTTCAAA-3′.

### Transcriptome analysis

Total RNA was extracted from the endometrial samples and then subjected to library construction. RNeasy Plus Micro Kit (Qiagen, Dusseldorf, Germany) was used to prepare total RNA. A NanoDrop spectrophotometer was used to assess the purity of RNA. The integrity of RNA was determined by Agilent 2100 Bioanalyzer. The sequencing library was prepared according to the instruction manual of a VAHTS total RNA-seq (H/M/R) Library Prep Kit for Illumina® (Vazyme, China). Transcriptome sequence analysis was carried out on Illuminahiseq2500 or Illumina Hiseq X10 platform by Vazyme (Nanjing, China). The differential expression of genes between the two groups was analyzed by DESeq algorithm.

### RNA extraction and qRT-PCR

Total RNA was extracted from cultured cells or tissues using TRIzol reagent (Invitrogen Life Technologies, Carlsbad, CA). The quality and purity of the RNA were detected by NanoDrop (Thermo Scientific, Waltham, MA, USA). The RNA was reverse-transcribed into cDNA using HiScript III RT SuperMix (Vazyme, China). Quantitative real-time PCR (qRT-PCR) assays were performed using SYBR qPCR Master Mix (Vazyme, China) on a LightCycler 480 machine (Roche, Pleasanton, CA, US). The relative expression levels of the mRNA were determined with the 2(−ΔCT) method. All primers used in this study are summarized in in Supplementary Table S2.

### Western blotting

Endometrial tissues or cultured HESCs were lysed in RIPA lysis buffer (Biosharp, China) added with protease inhibitor cocktail and phosphatase inhibitor cocktail (MedChemExpress, USA) for 30 min on ice. The supernatant was collected after centrifugation at 12,000×g for 20 min. Protein concentration was assessed with a Pierce BCA protein assay kit (Thermo Scientific, USA). The proteins were fractionated with SDS-PAGE gels, transferred to PVDF membranes (Bio-Rad, USA), and then blocked in 5% nonfat milk (Bio-Rad, USA) at room temperature. The membranes were incubated with appropriate primary antibodies overnight at 4 °C and then hybridized with a horseradish peroxidase-conjugated secondary antibodies for 1 h at room temperature. The bands were visualized with ECL solution (BioRad, USA). The antibodies used are summarized in Supplementary Table S3.

### Immunohistochemistry

Human endometrial tissues were fixed with 10% paraformaldehyde, embedded in paraffin, and cut into slices at 2 μm thickness. After paraffin removal and rehydration and blocking of endogenous peroxidase activity in 3% H_2_O_2_, slices were heat-mediated in universal antigen retrieval for 2 min. The slices were incubated with primary antibodies at 4 °C overnight and then incubated with HRP-conjugated secondary antibodies at room temperature for 8 min. Slides were exposed to DAB to visualize the antigen signals and counterstained with hematoxylin. After sealed with a neutral resin, the slides were then viewed under a microscope (DMi8, Leica, Germany).

### Statistical analysis

GraphPad Prism software (GraphPad Software, San Diego, CA, USA) was used to perform statistical analysis and all data are presented as means ± standard deviation (SD). One-way ANOVA followed by a Student-Newman-Keuls multiple comparisons test were used to compare three or more experimental groups. A Student’s t-test was used for comparisons of two experimental groups when the data were normally distributed. When the data were not normally distributed, a non-parametric test was used. Statistical significance was defined as *p* < 0.05.

## Results

### Expressed genes signature is different between normal and thin endometria

**Fig. 1:**
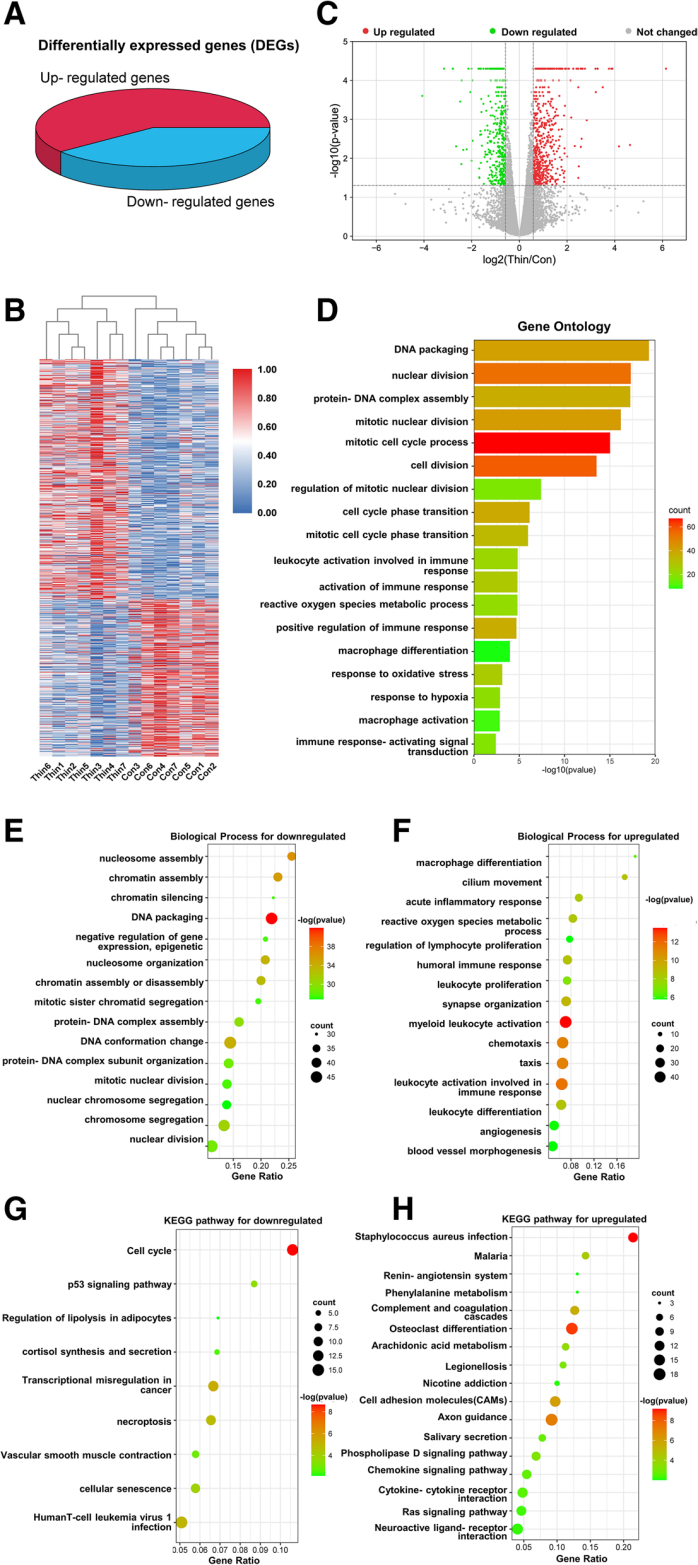
Identification and enrichment analysis of RNA-Seq differentially expressed genes between TE patients and controls. 3D pie plot (**A**), heatmap (**B**) and volcano plot (**C**) showing 521 up- and 353 downregulated differentially expressed genes (DEGs) (*p* < 0.05 and fold change > 1.5) in endometrium samples from thin endometrium (*n* = 7) and controls (*n* = 7). **D** Gene Ontology biological process analysis for all 874 DEGs in the thin endometrium. Gene Ontology biological process analysis and Kyoto Encyclopedia of Genes and Genomes (KEGG) pathway analysis for the down- (**E**) (**G**) and upregulated (**F**) (**H**) DEGs in the thin endometrium

A genome-wide mRNA expression analysis of endometria showed a total of 874 differentially expressed genes (DEGs) comprising 521 up- and 353 downregulated genes in TE compared to controls with the thresholds of *p*-value ≤0.05 and absolute value fold change ≥1.5 (Fig. 1A). Hierarchical clustering analysis of DEGs displayed that the gene expression patterns clustered separately after unsupervised clustering (Fig. 1B). These DEGs were also presented in a volcano plot (Fig. 1C).

Based on Metascape database (https://metascape.org/), Gene Ontology (GO) analysis of 874 DEGs showed that terms related to cell division and cell cycle had the largest enrichment proportion (Fig. 1D). These downregulated DEGs were significantly enriched in the process of cell division, cell cycle phase transition and other related functions (Fig. 1E), whereas annotations of upregulated genes were related to the activation of the inflammation, immune response and reactive oxygen species (Fig. 1F). With respect to GO cell component (CC) terms, the downregulated genes in TE were mainly present in the nucleus and cytoplasm, such as DNA packaging complex, whereas upregulated genes mainly existed in cytoplasm and extracellular components, such as extracellular matrix (Supplementary Fig. 1A). GO molecular function (MF) analysis showed that the main functions of these downregulated genes were involved in cyclin-dependent protein kinase regulator activity and the binding of cell components. Meanwhile, key predicted molecular functions of upregulated gene were related to channel activity and extracellular matrix structural constituent (Supplementary Fig. 1B).

Kyoto Encyclopedia of Genes and Genomes (KEGG) pathway analysis showed that the downregulated genes in the TE were mainly involved in cell cycle and p53 signaling pathway (Fig. 1G), while upregulated genes were enriched in immune inflammation and metabolic disorders, including arachidonic acid metabolism, cell adhesion molecules (CAMs), chemokine signaling pathway, and cytokine-cytokine receptor interaction (Fig. 1H). These results indicated that TE may have impaired cell proliferation.

### PPIs network analysis identifies PBK as a hub gene

**Fig. 2:**
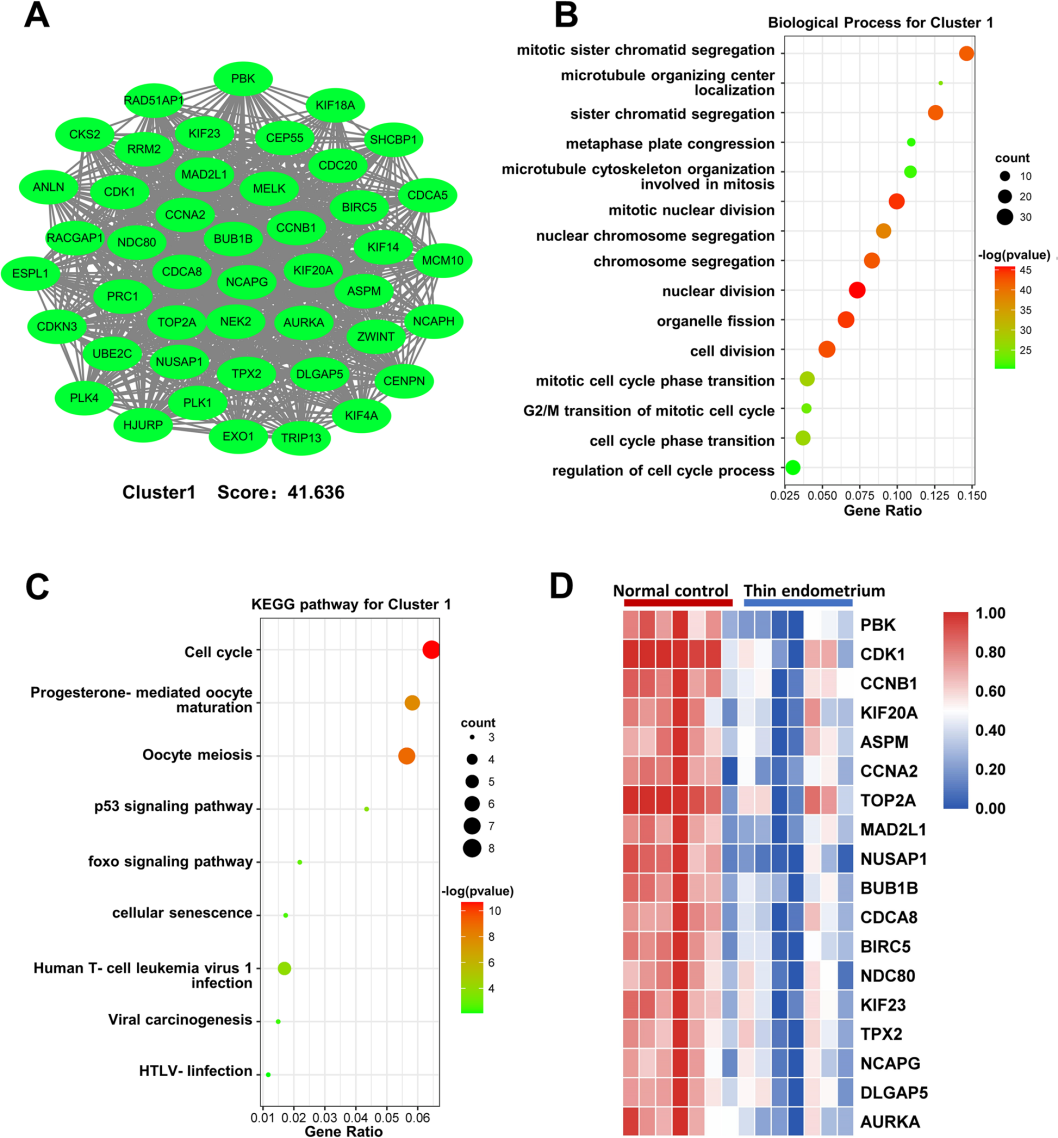
PPIs network construction and cluster analysis. **A** The top cluster 1 (score = 41.636) was derived from the protein-protein interactions network (PPIs) with molecular complex detection (MCODE) algorithm. Gene ontology analysis (**B**) and Kyoto Encyclopedia of Genes and Genomes pathway analysis (**C**) for the DEGs in cluster 1. **D** Heatmap showing 18 hub genes of cluster 1 selected by maximal clique centrality (MCC) ranking methods (the red nodes stand for upregulated genes; the green nodes represent downregulated genes)

The construction protein-protein interactions (PPIs) network was based on search tool for the retrieval of interacting genes/proteins (STRING) database (http://www.string-db.org/). A total of 874 DEGs data points were imported into the PPI network for analysis (minimum required interaction score ≥ 0.7), covering 3295 edges and 847 key nodes. Cytoscape shows the DEGs in the protein-protein interactions (PPIs) network (Supplementary Fig. 2). In addition, seven significant clusters with a score ≥ 7 were screened out via Cytoscape plugin molecular complex detection (MCODE) (Fig. 2A, Supplementary Fig. 3). Both cluster 1 and cluster 2 were composed entirely of downregulated genes, while clusters 3, 4, 5, 6 and 7 were mainly consist of upregulated genes.

To further investigate these different clusters, the GO and KEGG pathway analyses of the whole mRNAs included in each cluster were performed. DEGs of cluster 1 were involved in cell division, p53 and foxo signaling pathway (Fig. 2B, C). For the cluster 2, the most significant biological processes were nucleosome assembly and cell differentiation. The other five clusters, namely, clusters 3, 4, 5, 6 and 7, were enriched in immune response or inflammation process. Besides, cluster 4 and cluster 6 were also enriched in extracellular matrix organization and extracellular structure organization. The enrichment results of each cluster were presented in the Supplementary Table S4. According to the functional annotations and pathway enrichment analysis above, all of the seven clusters were able to be classified into two integrated functional modules artificially. Clusters 1 and 2 were termed abnormal cell proliferation and differentiation module, while clusters 3,4,5,6 and 7 were termed inflammation, immune and cell matrix metabolism disorders module. Cluster 1 had the highest MCODE score (41.636), indicating the key cluster. We used CytoHubba to screen the hub genes of this key cluster (the maximal clique centrality (MCC) ranking methods). The top 18 genes with the highest scores were considered as hub genes (Fig. 2D). The expressive abundance of PBK was significantly downregulated in TE group and was identified as the candidate gene.

### PBK is downregulated in the thin endometrium

**Fig. 3:**
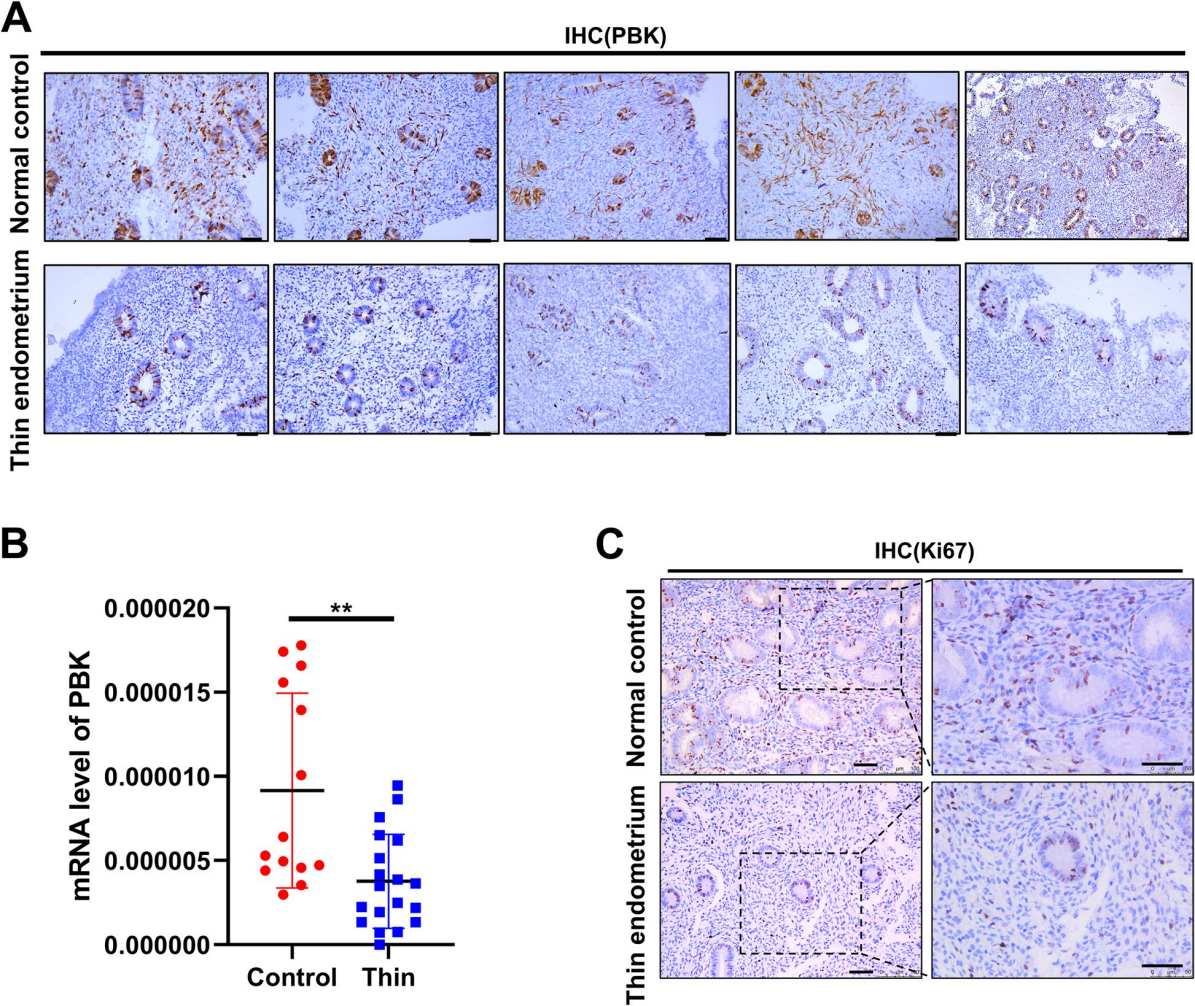
Downregulation of PBK in the thin endometrium. Representative PBK (**A**) and Ki67 (**C**) immunohistochemical staining in endometrial sections from controls (*n* = 5) and TE (*n* = 10) patients. Scale bar =50 μm. **B** The mRNA expression levels of PBK, relative to GAPDH, in endometria of controls (*n* = 14) and TE (*n* = 19) patients were measured by q-PCR. Result shows mean ± SEM. Data were analyzed with unpaired t-test.**P < 0.01 compared with the control group

Next, we validated the expression of PBK in endometria from TE patients and controls. Compared to controls, the mRNA level of PBK was decreased by about 2.43-fold in the thin endometria (Fig. 3B) and the protein level of PBK was also significantly decreased in the thin endometria. The localization of PBK by immunohistochemical staining showed that PBK was decreased particularly in the endometrial stromal cells (Fig. 3A), which was paralleled by decreased expression of Ki67, a marker of cell proliferation (Fig. 3C). These results suggested that the insufficient expression of PBK may be related to the poor proliferation of HESCs.

### Downregulation of PBK inhibits HESCs proliferation and promoted their apoptosis in vitro

**Fig. 4:**
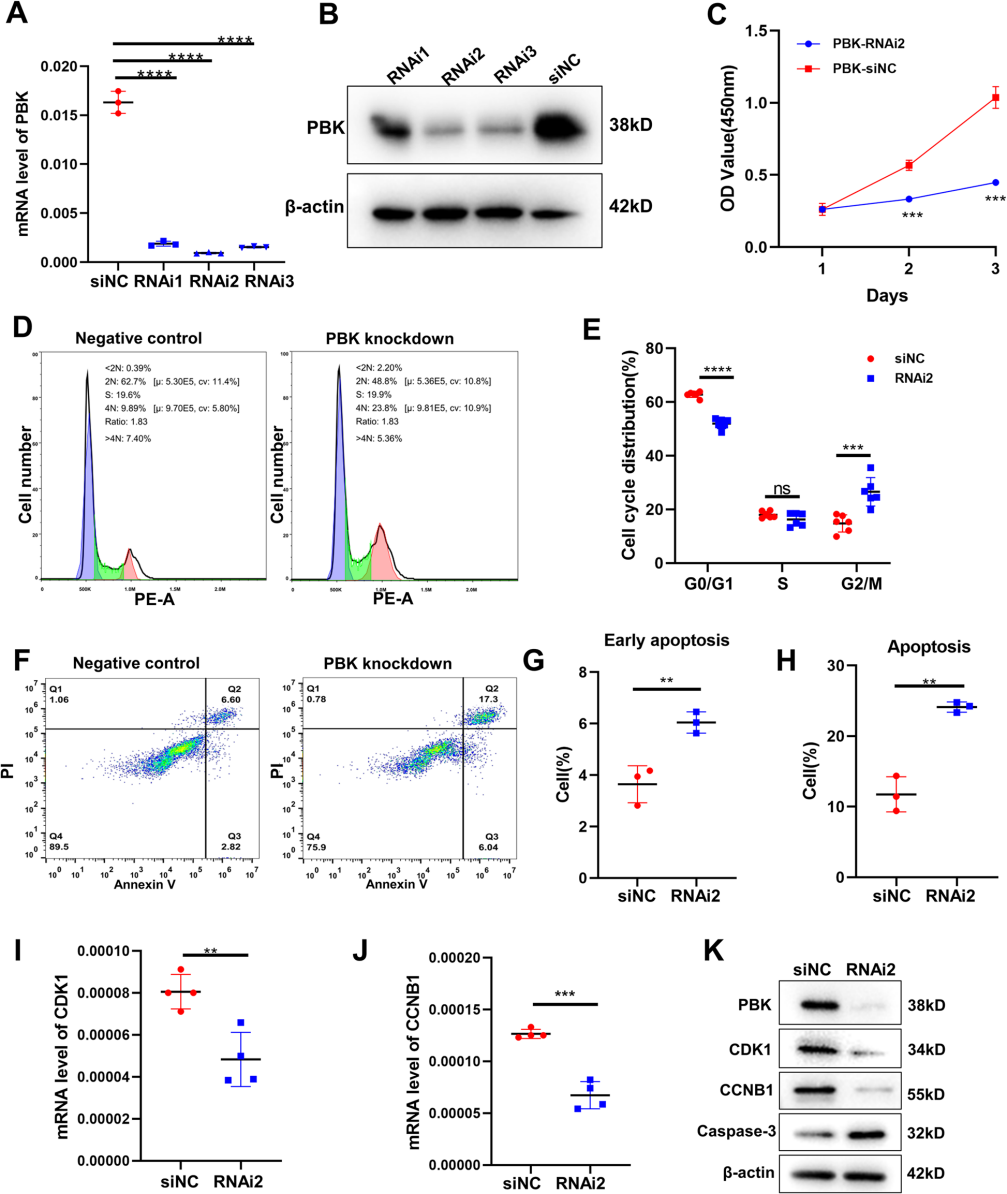
Effects of PBK knockdown on HESCs proliferation and apoptosis. PBK mRNA (**A**) and protein (**B**) levels in HESCs transfected with negative control (si-NC) or different small interfering RNAs of PBK (si-1, si-2, si-3) for 24 h (*n* = 3). **C** Cell proliferation after PBK knockdown in HESCs determined by the CCK-8 assay (*n* = 10). **D** and **E** Analysis of the effects of PBK knockdown (KD) on cell cycle distribution by flow cytometer (*n* = 6). **F**, **G** and **H** Flow cytometry showing apoptosis in HESCs with PBK KD (*n* = 3). **I**, **J** and **K** Effect of PBK KD on CDK1 and CCNB1 expression was assessed by qPCR and western blot (*n* = 4). (K) Effect of PBK KD on Caspase-3 expression was assessed by western blot. The results were representative of three independent experiments. All quantified data are presented as mean ± SEM; **P < 0.01, ***P < 0.0001, ****P < 0.0001

To investigate the effect of PBK on endometrial stromal cells’ proliferation, we constructed three small interference sequences of PBK (RNAi1 RNAi2 RNAi3) to knock down PBK in HESCs. Figure 4A and B showed that all three PBK RNAis could suppress PBK expression effectively. Then, we knocked down PBK (KD) in HESCs and found that cell proliferation decreased significantly in HESCs (Fig. 4C) and after 3 days of culture, HESCs proliferation was reduced to < 50% compared to the control group. Flow cytometry assay indicated that PBK KD induced G2/M phase arrest in the HESCs. PBK KD significantly decreased the percentage of G0/G1 phase cells and increased the percentage of G2/M phase cells (Fig. 4D, E), which was consistent with the results of GO analysis of Cluster 1 (Fig. 2B). CDK1 and CCNB1 are checkpoints for the cell cycle progression of G2/M phase, and are also two of 18 HUB genes in MCODE Cluster 1. Western blotting revealed that the protein levels of PBK and CCNB1 decreased significantly in HESCs with PBK KD (Fig. 4K). Furthermore, the flow cytometry assay revealed that PBK KD increased apoptosis levels of HESCs by 1.05-fold (Fig. 4F, G and H). Western blot analysis showed that the protein levels of Caspase-3 were upregulated in the si-PBK group compared with si-NC group (Fig. 4K). These results directly demonstrated that the insufficient expression of PBK played an important role in suppressing the proliferation and promoting the apoptosis of HESCs.

### PBK inhibits HESCs proliferation via p53 signaling pathway

**Fig. 5:**
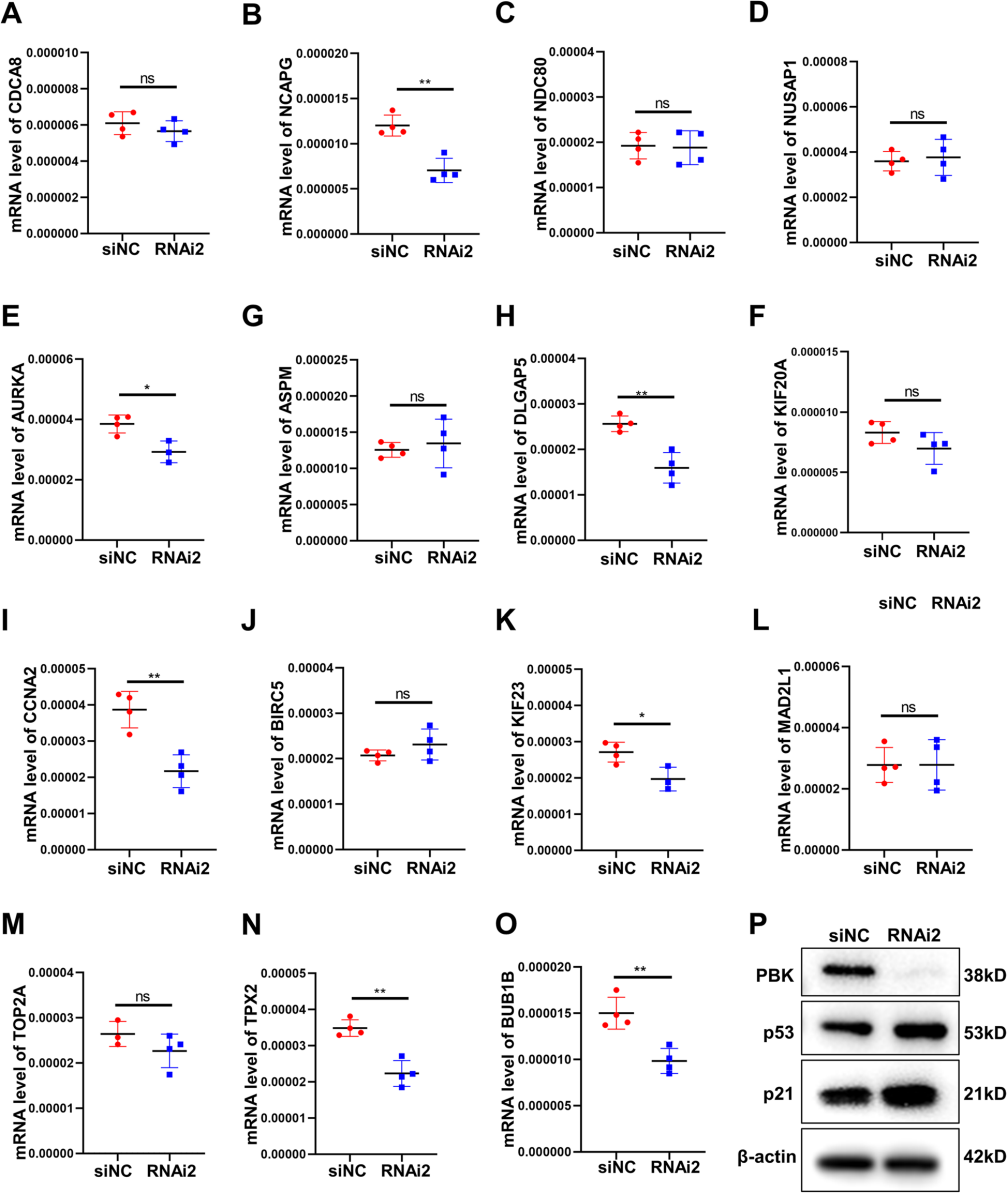
Inhibitory effects of PBK on HESCs proliferation via p53 signaling pathway. **A**-**O** The mRNA levels of other hub genes were examined by qPCR in HESCs transfected with siPBK-2 after 24 h (*n* = 4). **P** The protein levels of p53 and p21 with PBK KD for 24 h. The results were representative of three independent experiments. All quantified data are presented as mean ± SEM; *P < 0.05, **P < 0.01

To explore how PBK performs the functions above, we first verified the effect of silencing PBK on other hub genes in Cluster 1. As the results showed, the mRNA expression levels of most hub genes (9/17) decreased significantly after transfection with si-PBK, which suggested the key role of PBK in Cluster 1 (Fig. 4I and J, Fig. 5A-O). Previous KEGG analysis showed that p53 and foxo signaling pathways were two significant enrichment pathways in cluster 1 (Fig. 2C). We thus analyzed whether PBK KD may affect HESCs proliferation via p53 signaling pathway in vitro. In the PBK KD HESCs, western blot analysis showed that the expression of p53 was increased, and its downstream target p21 was enhanced more strongly (Fig. 5P). However, inhibition of PBK did not interfere with the expression of FOXO1 and ERK in HESCs (Supplementary Fig. 4A, B). These results suggested that the p53/p21 signaling axis is a key signaling pathway for PBK KD to inhibit cell proliferation and promote cell apoptosis.

### PBK expression is inhibited by oxidative stress, hypoxia and inflammatory factors

**Fig. 6:**
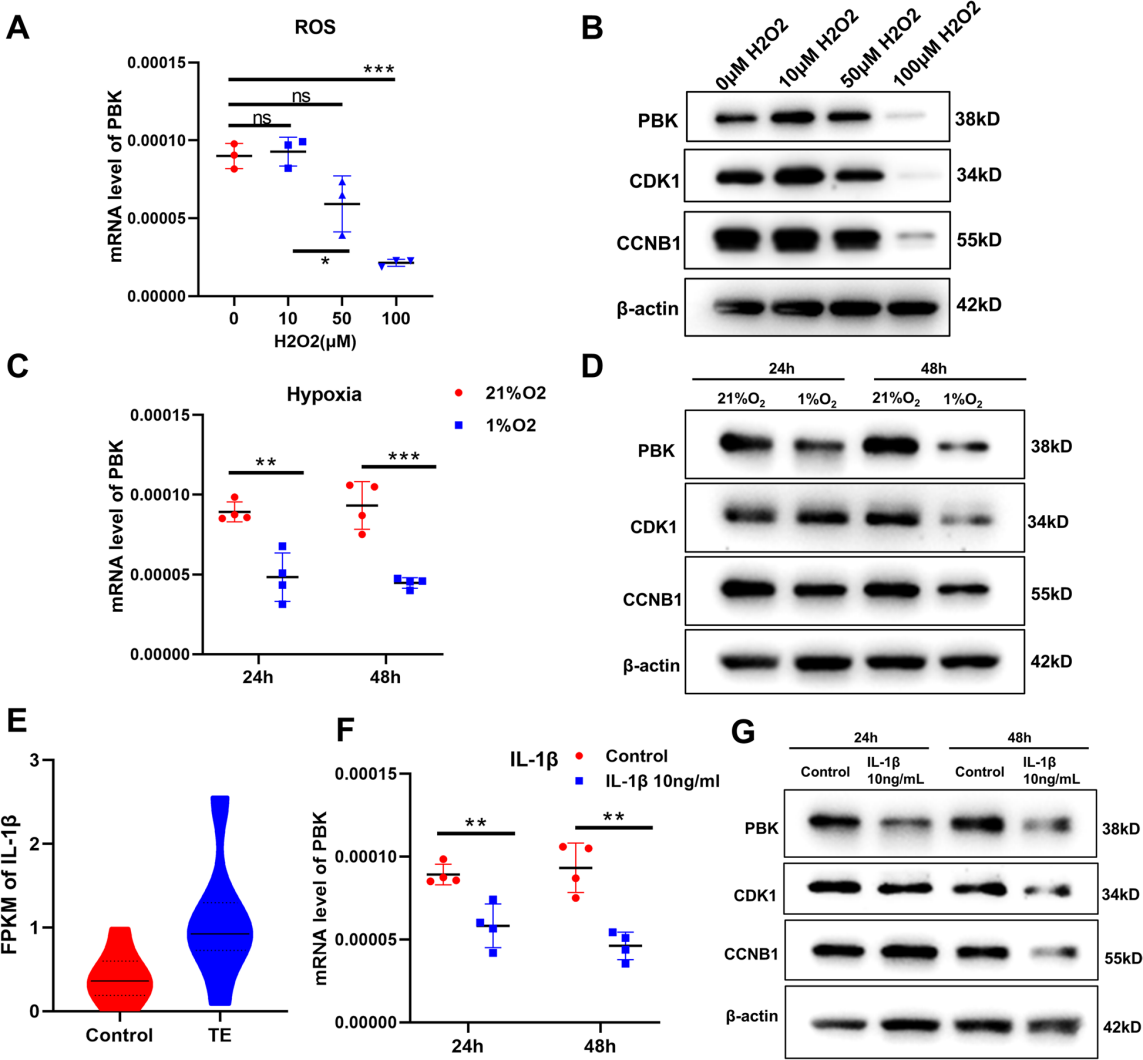
The contribution of oxidative stress, hypoxia and inflammatory factors to PBK downregulation. **A** The mRNA level of PBK in HESCs treated with different concentrations of hydrogen peroxide for 48 h (*n* = 3). **B** The protein levels of PBK, CDK1 and CCNB1 were detected by western blot after 48 h. **C** The mRNA level of PBK in HESCs under hypoxia condition with 1% O_2_ at different time points (*n* = 4). **D** The protein levels of PBK, CDK1 and CCNB1 were detected by qPCR after 24 h or 48 h. **E** RNA-seq results showed that IL-1β (10 ng/mL) in endometrium samples from TE patients was upregulated compared with that in controls. Fold change = 2.45, *P*-value = 0.0398. **F** The mRNA level of PBK in HESCs stimulated with 10 ng/ml IL-1β at different time point (*n* = 4). **G** The protein levels of PBK, CDK1 and CCNB1 were detected by western blot after incubation with 10 ng/ml IL-1β for 24 h or 48 h. The results were representative of three independent experiments. All quantified data are presented as mean ± SEM; **P < 0.01, ***P < 0.001. TE: thin endometrium

Since the result of RNA-seq suggested that there existed inflammatory activation and disturbance of reactive oxygen species in endometria of TE patients (Fig. 1F), we explored the association of inflammation and ROS with the expression of PBK in HESCs in vitro. We treated HESCs with different doses of H_2_O_2_ (0, 10,50 and 100 μM) for 48 h and found that H_2_O_2_ at 100 μM reduced the expression level of PBK by 76.2% (Fig. 6A and B). At the same time, the protein levels of CDK1 and CCNB1 were also decreased markedly (Fig. 6B). Next, we investigated the effect of hypoxia on PBK expression in HESCs. qPCR showed that the mRNA level of PBK was decreased by 51.9% in HESCs after exposure to hypoxia (1% O_2_), and the protein levels of PBK, CDK1 and CCNB1 were decreased significantly with the prolongation of hypoxia (Fig. 6C and D). Since RNA-seq analysis showed that IL-1β expression was significantly upregulated (approximately 2.63 times) in the endometria of TE patients (Fig. 6E), we studied the effect of IL-1β on the expression of PBK in vitro. The results showed that mRNA and protein expression levels of PBK were decreased by 50.5% after the HESCs stimulated with IL-1β (10 ng/mL) for 48 h, and the expression levels of CDK1 and CCNB1 were also decreased (Fig. 6F and G). Then we proved that the expression of PBK in HESCs could also be inhibited by TNFα (20 ng/mL; decreased by 40.1%) and TGFβ1 (10 ng/mL; decreased by 56.1%) (Supplementary Fig. 5A-D). Taken together, these results suggested that oxidative stress, hypoxia and inflammatory factors can regulate PBK expression.

## Discussion

The objective of this study was to investigate the transcriptional landscape of late-proliferative phase endometria in TE patients and scan a set of genes relevant to TE. We found that the late-proliferative phase endometria of TE patients showed a significant inhibition of cell proliferation, which was related to the insufficient expression of PBK. Overactivation of inflammation and disorders of reactive oxygen species negatively modulated the expression of PBK and played an important role in the development of TE.

The human endometrium is a highly self-renewing tissue that is periodically shed, repaired, regenerated and remodeled, under the coordination of estrogen and progesterone, in preparation for embryo implantation [18]. Recently published studies analyzed the gene expression profiles between proliferative and mid-secretory endometria in fertile women, DEGs upregulated in the proliferative endometrium were significantly enriched in cell cycle, whereas downregulated DEGs were significantly enriched in immune system process and immune response [19]. The proliferative phase, which occurs following menstruation and precedes ovulation, is marked by the active growth of several cell types including HESCs, epithelial, and endothelial cells [20], and by ovulation, the average thickness of the endometrium reached about 12 mm, while during the luteal phase, endometrial growth tends to plateau and turned to differentiation [21]. These studies suggest that the proliferative phase is more critical for endometrial thickening.

In the present study, the most predominant functional enrichment in TE was negative regulation of cell cycle and proliferation in late proliferative phase. We constructed the PPIs network and identified seven clusters most likely associated with the pathogenesis of TE. PBK was identified as the hub of the key cluster, and its expression level was significantly reduced in the TE group, especially in stromal cells. PBK, a novel serine/threonine kinase, is expressed in proliferative cells and tissues and plays an important role in cell cycle [22], apoptosis [23] and spermatogenesis [24]. Knockdown of PBK inhibits the proliferation of human epidermal keratinocytes and neural progenitor cells [25, 26]. In the present study, we showed that the inhibition of PBK could suppress the proliferation and promote the apoptosis of the HESCs, which implied that decreased expression of PBK during the late-proliferative phase was related to inhibiting the endometrial thickening. Our study showed that upregulated differentially expressed transcripts of TE patients were significantly associated with overactivated immune, inflammatory response and oxidative stress. These representative genes, interleukin 1 beta (IL-1β) and tumor necrosis factor alpha (TNFα) induced genes could inhibit PBK expression, which was agreed with the increased expression of IFN-γ and TNFα in TE in previous report [27]. These results suggest that there is an aberrantly pro-inflammatory environment in the late-proliferative phase endometrium of TE patients, which is related to the impaired endometrial thickness. In addition, our RNA-seq results also showed that the expression of hypoxia inducible factor 3 alpha subunit (HIF3A) was significantly upregulated in the endometria of patients with TE and previous pathophysiologic study showed that patients with TE have a higher level of blood flow impedance of the uterine radial artery and significantly fewer endometrial blood vessels [27]. Thus, we can infer that the endometria of TE patients have insufficient supply of nutrition and oxygen, which may be related to the occurrence of TE, supported by the evidence that prolonged hypoxia resulted in the downregulation of PBK, CDK1 and CCNB1 in HESCs.

Compared with other studies of TE, our study is the first to focus on the late-proliferative phase endometria in TE patients, and identifies several potential factors associated with the pathogenesis of TE, which provide a novel perspective for the extensive comprehension of the disease. We found a hub gene PBK and validated its important role in the proliferation of endometrial stromal cells. However, our study also had some limitations. First, all TE patients enrolled were directly related to repeated uterine curettage so the findings may not explain the other etiological thin endometrium [28]. In addition, the role of PBK in the pathogenic mechanism of TE needs further experiment in vivo. Finally, considering the complexity of the disease, the functions of other hub genes and clusters also need to be studied .

## Conclusions

In conclusion, our study showed the transcriptional landscape of late-proliferative phase endometria in TE, and found a set of genes relevant to TE. We demonstrated that the expression of PBK was downregulated and showed that insufficient PBK contributed to thin endometrium by inhibiting HESCs proliferation and promoting their apoptosis. These findings will help to further explore the new therapeutic strategies of TE.

## Supplementary Information

**Additional file 1: Supplementary Figure 1**. Enrichment analysis of differentially expressed genes. Functional enrichment analysis of the up- and downregulated DEGs, including GO category cellular components (A), and molecular function (B). Red stand for upregulated genes, blue represent downregulated genes. **Supplementary Figure 2.** PPIs Network Construction. The PPIs network of DEGs was constructed by STRING and Cytoscape. (The red nodes stand for upregulated genes; the green nodes represent downregulated genes). **Supplementary Figure 3.** PPIs Network Cluster Analysis. (A) The top clusters 2–6 in MCODE analysis of DEGs (The red nodes stand for upregulated genes; the green nodes represent downregulated genes). **Supplementary Figure 4**. Effects of PBK knockdown on foxo and ERK signaling pathways. The protein level of FoxO1(A), p-ERK and t-ERK (B) in HESCs transfected with siPBK-2 after 24 h. The results were representative of three independent experiments. **Supplementary Figure 5**. Effects of TNFα and TGFβ1 on PBK expression. The mRNA and protein levels of PBK in HESCs stimulated with 20 ng/ml TNFα (A and B) or 10 ng/ml TGFβ1 (C and D) after 24 h or 48 h (*n* = 4). The results were representative of three independent experiments. All quantified data are presented as mean ± SEM; **P < 0.01, ***P < 0.001. **Table S1**. Clinical information of all patients and controls; **Table S2**. Sequences of primers; **Table S3**. Antibodies used for immunohistochemistry (IHC) and western blotting (WB); **Table S4**. Cluster2–7 enrichment analysis.

## Data Availability

The data that support the findings of this study are available from the corresponding author upon reasonable request.

## Abbreviations

TE: Thin endometrium
HESCs: Human endometrial stromal cells
PBK: PDZ-binding kinase
PPIs: Protein-protein interactions network
DEGs: Differentially expressed genes
qRT-PCR: Quantitative real time polymerase chain reaction
GO: Gene Ontology
KEGG: Kyoto Encyclopedia of Genes and Genomes
CC: GO cell component
MF: GO molecular function
MCODE: Molecular complex detection
MCC: Maximal clique centrality
KD: Knockdown
RNA-seq: RNA sequencing
CCK-8: Cell Counting Kit-8
IHC: Immunohistochemistry
STRING: Search Tool for the Retrieval of Interacting Genes
