# Supplementary material for: Down-regulation of PBK inhibits proliferation of human endometrial stromal cells in thin endometrium
Source: Reprod Biol Endocrinol. 2022 Feb 2;20:25. doi: 10.1186/s12958-022-00903-8 (PMC8809007; doi:10.1186/s12958-022-00903-8)
Supplement: Supplementary file 1 — Additional file 1: Supplementary Figure 1. Enrichment analysis of differentially expressed genes. Functional enrichment analysis of the up- and downregulated DEGs, including GO category cellular components (A), and molecular function (B). Red stand for upregulated genes, blue represent downregulated genes. Supplementary Figure 2. PPIs Network Construction. The PPIs network of DEGs was constructed by STRING and Cytoscape. (The red nodes stand for upregulated genes; the green nodes represent downregulated genes). Supplementary Figure 3. PPIs Network Cluster Analysis. (A) The top clusters 2–6 in MCODE analysis of DEGs (The red nodes stand for upregulated genes; the green nodes represent downregulated genes). Supplementary Figure 4. Effects of PBK knockdown on foxo and ERK signaling pathways. The protein level of FoxO1(A), p-ERK and t-ERK (B) in HESCs transfected with siPBK-2 after 24 h. The results were representative of three independent experiments. Supplementary Figure 5. Effects of TNFα and TGFβ1 on PBK expression. The mRNA and protein levels of PBK in HESCs stimulated with 20 ng/ml TNFα (A and B) or 10 ng/ml TGFβ1 (C and D) after 24 h or 48 h (n = 4). The results were representative of three independent experiments. All quantified data are presented as mean ± SEM; **P < 0.01, ***P < 0.001. Table S1. Clinical information of all patients and controls; Table S2. Sequences of primers; Table S3. Antibodies used for immunohistochemistry (IHC) and western blotting (WB); Table S4. Cluster2–7 enrichment analysis. [file 12958_2022_903_MOESM1_ESM.zip › Table S1.docx]

**Supplementary Table 1: Clinical information of all patients and controls.**

| Items | Control | Thin endometrium | *P* value |
| --- | --- | --- | --- |
|  | (n = 26) | (n = 36) |  |
| Age (year) | 31.58 ±0.86 | 33.53 ±0.63 | > 0.05 |
| Times for D&C | 1.04 ±0.24 | 2.67 ±0.27 | < 0.0001 |
| Times for TCRA | 0 | 1.81 ±0.15 | < 0.0001 |
| Maximum preoperative EMT(mm) | 9.83 ±0.23 | 4.87 ±0.14 | < 0.0001 |
| Times of ET cancellation | 0 | 1.05 ±0.24 | < 0.001 |

D&C: dilation and curettage; TCRA: transcervical resection of adhesion; EMT: endometrial thickness; ET: embryo transfer
